# Numerical Modelling of the Nonlinear Shear Creep Behavior of FRP-Concrete Bonded Joints

**DOI:** 10.3390/ma16020801

**Published:** 2023-01-13

**Authors:** François Soleilhet, Marc Quiertant, Karim Benzarti

**Affiliations:** 1 EDF Lab Les Renardières, F-77250 Moret-Loing-et-Orvanne, France; 2 Matériaux et Structures (MAST) Department, Expérimentation et Modélisation pour le Génie Civil et Urbain (EMGCU), University Gustave Eiffel, F-77447 Marne-la-Vallé, France; 3 Lab Navier, University Gustave Eiffel, Ecole Nationale des Ponts et Chaussées (ENPC), Centre National de la Recherche Scientifique (CNRS), F-77447 Marne la Vallée, France

**Keywords:** epoxy adhesive, FRP-concrete bonded joints, nonlinear creep, finite element modelling

## Abstract

This paper presents a numerical investigation of the shear creep behavior of the adhesive joint in concrete structures strengthened by externally bonded fibre-reinforced polymers (FRP) composites. Based on experimental data collected in a previous study, creep constitutive equations were developed for the adhesive layer and implemented into a finite element code. The proposed model extends the classical one-dimensional formulation of Burgers creep model to a fully 3D model and introduces the nonlinearity of the model parameters. This numerical approach was first used to simulate the nonlinear creep behavior of bulk epoxy samples; it was then extended to predict the nonlinear creep response of the FRP-concrete interface in double lap shear specimens. Globally, a fair agreement was obtained between numerical results and experimental evidences. As a main result, it was found that creep induces a redistribution of the interfacial shear stress along the FRP-concrete lap joint, leading both to a stress relaxation near the loaded end of the adhesive joint and to an increase in the effective transfer length.

## 1. Introduction

Externally Bonded (EB) Fiber Reinforced Polymer (FRP) strengthening systems have been widely adopted for the rehabilitation of Reinforced Concrete (RC) structures since the late 1990’s. The effectiveness of this retrofitting method has been clearly demonstrated in the literature [1,2,3,4,5,6] and several well-established international design guidelines are now available to practitioners with a view to ensuring proper field implementation [7,8,9,10,11,12]. In EB-FRP applications, service loads are transferred from the concrete structure to the FRP reinforcement through the adhesive joint, which deforms mainly under shear stress. Due to its inherent viscoelastic nature, the polymer adhesive layer may exhibit substantial creep deformation when subjected to sustained shear load, hence affecting the stress transfer process between the host structure and the FRP material [13,14,15,16]. This creep effect is possibly emphasized by the evolution of the adhesive properties (loss of stiffness) due to environmental ageing under service conditions [17,18]. In this context, the long-term performance of the structural retrofit strongly relies on the delayed behavior of the adhesive joint. Therefore, knowledge of the time dependent response of the bulk polymer adhesive and the FRP-concrete adhesive bond under creep load is of great importance for safety issues and for optimizing the maintenance strategy as well. It becomes even crucial in the case of prestressed FRP systems, where delayed viscous effects of the adhesive bond may result in critical prestress losses [19,20].

The viscoelastic response of bulk polymer materials has been widely investigated in the literature, through both experimental studies and theoretical approaches. Experimental works rely most often on tensile tests at constant load levels [16,21,22,23] although few authors have developed specific setups to investigate the creep behavior of bulk polymers subjected to sustained shear loading [24]. Differently, the creep behavior of the adhesive joints in FRP-concrete assemblies is less documented. In general, it is admitted that adhesive joints behave differently from the bulk polymer material, as they are subjected to complex stress fields [25,26]. For this reason, the creep response of the adhesive layer is often directly investigated by performing shear tests on FRP-concrete assemblies [13,27,28]. Nevertheless, several researchers have shown that the delayed response of FRP-concrete specimens can still be accurately described by identifying in a first stage the creep behavior law of the bulk adhesive, and then introducing this law into a finite element (FE) model of the FRP-concrete assembly. For instance, Zehsaz et al. [29] carried out uniaxial tensile creep tests on bulk epoxy samples, which allowed them to identify the parameters of a rheological model derived from Maxwell and Zener’s model [29]. This model was then used to predict the creep response of double shear joints using the FE method, and numerical results showed good agreement with experimental data. A similar approach was adopted by Houhou et al. [17]. These authors performed short-term tensile creep tests on bulk adhesive samples at several temperatures and at different stress levels, and they used these experimental data to build the creep compliance master curve according to the time-temperature superposition principle (TTSP). A rheological Burgers model was then calibrated from this master curve, which allowed to describe the nonlinear creep response of the bulk adhesive. Finally, this behavior law was successfully implemented in a FE model to predict the creep response of FRP-concrete bonded assemblies. Besides, Houachine et al. [16] proposed an analytical model to describe the long-term load-bearing capacity of concrete beams strengthened with EB-FRPs, which includes the creep contributions of all component materials (i.e., concrete, adhesive and FRP). A combined cohesive-bridging zone model was then proposed to model both the crack-processing zone near the interface (cohesive zone model) and the particle-interlocking region (bridging zone model). To predict the long-term response of the FRP-concrete interface, the fracture energy was kept constant in each zone and at each step of the calculation. After calibration of the bond-slip model with experimental results from the literature describing the instantaneous response of the FRP-concrete interface, long-term simulations were compared to those of Houhou et al. [17], showing good agreement.

Hadjazi et al. [30] also studied the long-term behavior of RC beams strengthened by EB-FRPs, but with a particular focus on the time evolution of shear stresses induced by flexural cracks along the adhesive joint. In continuity with previous works [16,31], these authors considered the creep behaviors of all the component materials of the retrofitted beam to develop an original viscoelastic model, in which the influence of environmental conditions are introduced through adjusted creep factors depending on the relative humidity. It is noteworthy that Eurocode 2 [32] suggests the construction of a creep function based on the creep behavior laws of all component materials of the bonded assembly (concrete, adhesive and FRP). Following a cohesive zone model approach and considering the total energy constant, the approach of Hadjazi et al. [30] was found effective to analyze the creep response of a FRP-plated RC beam, including the debonding stage of the FRP plate. In most studies from the literature, unidimensional formulations of the rheological creep models are proposed. This approach is relevant for many strengthening configurations using FRPs, like flexural strengthening of longitudinal beams, where the predominant load in the bonded FRP reinforcement is uniaxial tension. It has also proven to be effective in several FE analysis [33]. However, 1-D formulation is less appropriate in the case of structures with complex geometries, where non-planar bonding surfaces are submitted to 2-D or 3-D stress fields [34].

In this study, a simple fully 3-D formulation of the creep behavior of the adhesive layer is proposed, that considers the stress-dependency of model parameters. The main originality of this approach lies in the proposal of a multi-axial creep model developed in a 3D tensor formalism. In this framework, the nonlinearity of the adhesive creep is managed by correlating the model parameters with the mechanical stress state of the polymer material. This approach could also be enriched at a further stage by taking into account the dependency of the adhesive creep upon hygro-thermal conditions. A second originality of the model is the use of the constitutive law generator MFront, which allows a portability of the behavior law between different FE codes (ABAQUS, CAST3M, Code_Aster,…) without further development. The proposed model is then used to predict the creep behavior of double shear specimens, and the numerical results obtained are compared to those previously obtained by Houhou et al. [17] for the same test configuration [17,35].

## 2. Nonlinear Creep Model

When an FRP retrofitted RC structure is exposed to sustained load, concrete creep occurs in the compression zone, FRP laminates are subjected to tensile creep and the adhesive joint is rather subjected to shear creep. In this paper, we will focus on the creep behavior of the adhesive layer, as this latter is considered to bring the highest contribution to the delayed response of the overall assembly near the loaded end of the adhesive joint. Moreover, in actual repair works, the RC beam has generally been subjected to sustained load for a long period before application of the FRP strengthening system and, thus, concrete and steel have already experienced significant part of their creep strains. However, the proposed model could be applied (after adequate calibration) to all the component materials of the FRP-strengthened RC structure. In the following section, the key characteristics of the adhesive joint are discussed. As proposed in [35], an uncracked concrete substrate is considered in order to simplify the analysis and highlight the global effects of the adhesive creep.

Although creep of polymers is known to be dependent on time, stress, and environmental conditions [36], the proposed modelling of the time-dependent strain in a viscoelastic 3D framework is developed accounting for material nonlinearities and stress state dependency, but it will not take into consideration the effects of temperature and relative humidity. Therefore, the application of this model is restricted to an isothermal condition in the temperature range below the glass transition temperature ($T_{g}$) of the polymer adhesive.

### 2.1. Constitutive Equations

**Figure 1:**
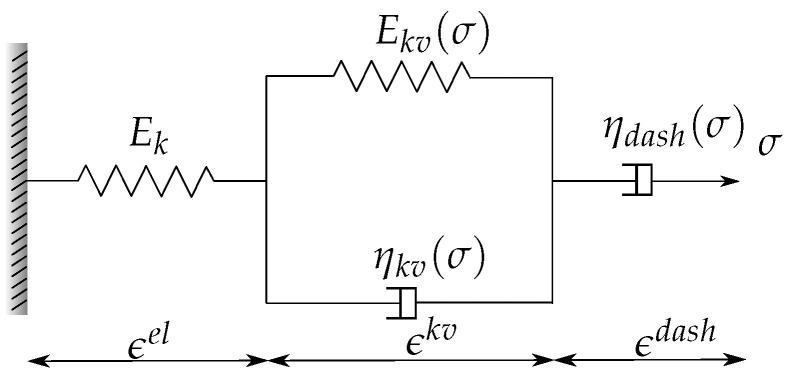
1D schematic representation of Burgers material.

For the sake of clarity, the one-dimensional Burgers material, which is the base of the present model, is depicted (Figure 1). However, all the following equations are developed in a three-dimensional framework. Burgers material is composed of three main blocks organized in series: an elastic material (a Hookean spring exhibiting a modulus of elasticity E${}_{k}$), followed by a Kelvin–Voigt solid (with modulus of elasticity E${}_{kv}$ of the spring and viscosity $\eta_{kv}$ of the dashpot), which is finally connected to a linear dashpot ($\eta_{dash}$).

Therefore, the total strain rate is decomposed into three terms:

(1) $${\dot{\epsilon }}^{to}={\dot{\epsilon }}^{el}+{\dot{\epsilon }}^{kv}+{\dot{\epsilon }}^{dash}$$

where the dot on top of strain stands for time derivative. ϵ, $\epsilon^{el}$, $\epsilon^{kv}$ and $\epsilon^{dash}$ are, respectively, the tensors of the total, the elastic, the delayed elastic strain of the Kelvin–Voigt solid and the viscous strain of the Maxwell dashpot.

#### 2.1.1. Elastic and Viscous Strains

From Equation (1) it can be concluded that the rate of total strain ($\dot{\epsilon }$) is divided into an elastic part (${\dot{\epsilon }}^{el}$) and a viscous part (${\dot{\epsilon }}^{v}$).

(2) $${\dot{\epsilon }}^{to}={\dot{\epsilon }}^{el}+{\dot{\epsilon }}^{v}$$

The elastic part of the strain rate, corresponding to instantaneous strain response, is given by Hooke’s law:

(3) $${\dot{\epsilon }}^{el}=q_{k}C_{v}:\dot{\sigma }$$

where “:” stands for double dot product, $q_{k}$ (${\mathrm{Pa}}^{-1}$) the elastic compliance ($q_{k}$ = 1/$E_{k}$ with $E_{k}$ (Pa) the elastic stiffness) and $C_{v}$ the unit tensor of elastic compliance (fourth order tensor) which is defined in Appendix A (written in Voigt notation).

The evolution of the viscous part of the strain (${\dot{\epsilon }}^{v}$) is the sum of the strain evolution of the Kelvin–Voigt material described by a second order equation (Equation (4b)) and the evolution of the strain in the dashpot governed by Equation (4c). Thus, the three equations below govern the evolution of the model viscous part.

(4a) $${\dot{\epsilon }}^{v}={\dot{\epsilon }}^{kv}+{\dot{\epsilon }}^{dash}$$

(4b) $$\tau_{kv}{\dot{\epsilon }}_{v}^{kv}+\epsilon_{v}^{kv}=q_{kv}\left(\sigma \right)\;C_{v}:\sigma \;\mathrm{with}\;q_{kv}\left(\sigma \right)=\frac{1}{E_{kv}\left(\sigma \right)}$$

(4c) $${\dot{\epsilon }}^{dash}=q_{dash}\left(\sigma \right)\;C_{v}:\sigma \;\mathrm{with}\;q_{dash}\left(\sigma \right)=\frac{1}{\eta_{dash}\left(\sigma \right)}$$

where $q_{kv}$ (${\mathrm{Pa}}^{-1}$) and $E_{kv}$ (Pa) are, respectively, the creep compliance and elastic modulus of the Kelvin–Voigt unit, $\tau_{kv}$ (s) the retardation time equal to $\eta_{kv}/E_{kv}$, $q_{dash}$ (${\mathrm{Pa}}^{-1}$) and $\eta_{dash}$ (Pas) the creep compliance and the dynamic viscosity associated to the linear dashpot part. The creep Poisson’s ratio of the polymer is considered constant over time [37,38] and it is taken equal to the elastic Poisson’s ratio (ν) due to the lack of information regarding transverse strain. Thus, the relaxation modulus in the shear direction ($G_{kv}$) can be calculated from the relaxation modulus in axial direction ($E_{kv}$) according to Equation (5).

(5) $$G_{kv}=\frac{E_{kv}}{2(1+\nu )}$$

#### 2.1.2. Nonlinearity

Experimental evidences from the literature [17,39,40] have demonstrated the nonlinearity of the adhesive creep behavior. To manage this stress dependency, model parameters such as Kelvin–Voigt solid’s stiffness and dynamic viscosity parameters ($\eta_{dash}$ and $\eta_{kv}$) are considered dependent upon applied stress (σ). As suggested by Majda and Skrodzewicz [41], the nonlinearities are introduced by exponential function (Equations (6a) and (6b)).

(6a) $$E_{kv}\left(\bar{\sigma }\right)=E_{kv}^{0}\exp\left(a_{kv}\bar{\sigma }\right)$$

(6b) $$\eta_{i}\left(\bar{\sigma }\right)=\eta_{j}^{0}\exp\left(b_{j}\bar{\sigma }\right)$$

with $E_{kv}^{0}$ (Pa), $\eta_{j}^{0}$ (Pas), respectively, the initial stiffness and the initial dynamic viscosity, $a_{kv}$ and $b_{j}$ both in (${\mathrm{Pa}}^{-1}$) two scalars to account for the material nonlinearities (the index *j* refers to kv or dash depending on the case), and finally $\bar{\sigma }$ (Pa) is a scalar value called the effective stress. To determine the effective stress, a linear combination of shear ($\sigma_{e}$) and hydrostatic ($\sigma_{m}$) components of stress tensor (Equation (7)) is used as formulated by Dean [39]. This combination is an analogy to the Drucker–Prager yield criterion.

(7) $$\begin{array}{cc} \bar{\sigma } & =\frac{\left(\alpha +1\right)}{2\alpha }\sigma_{e}+\frac{3(\alpha -1)}{2\alpha }\sigma_{m}\\ \sigma_{e} & ={\left[\frac{1}{2}\left[{\left(\sigma_{I}-\sigma_{II}\right)}^{2}+{\left(\sigma_{II}-\sigma_{III}\right)}^{2}+{\left(\sigma_{I}-\sigma_{III}\right)}^{2}\right]\right]}^{\frac{1}{2}}=\sqrt{3J_{2}}\\ \sigma_{m} & =\frac{1}{3}\left(\sigma_{I}+\sigma_{II}+\sigma_{III}\right)=\frac{I_{1}}{3} \end{array}$$

with $\sigma_{I}$, $\sigma_{II}$, $\sigma_{III}$ the principal stresses of the stress tensor, $J_{2}$ the second principal invariant of the stress tensor and α a scalar parameter. From Equation (7), it can be concluded that under a uniaxial tensile stress $\sigma_{t}$, the effective stress is equal to $\sigma_{t}$, while under a uniaxial compressive stress $\sigma_{c}$, the effective stress is equal to $\sigma_{c}/\alpha$. One should note that for low stress levels, the compliance curves obtained in tension or in compression look very similar. However, under higher stresses where material behaviors are expected to be nonlinear, differences may occur between tension and compression loadings (see for instance the study of Dean [39]). In this study, considering that there is no information regarding creep in compression for the experimental test modelled hereafter, α is always assumed equal to 1. Nevertheless, it is possible to consider tension–compression asymmetry through this parameter.

### 2.2. Implementation

MFront constitutive law generator [42,43,44] available in the FE software Code_Aster was used to implement the nonlinear creep model. Time integration was carried out using an implicit method based on a θ-scheme.

#### 2.2.1. Implicit Scheme

For the aforementioned θ-scheme, the state variables to account for are the elastic strain $\epsilon^{e}$ and the viscous strains $\epsilon^{kv}$ and $\epsilon^{dash}$. The equations which govern the evolution of these state variables are determined from the increments of the different strains. To obtain these strain increments, time is subdivided into discrete instant $t_{i}$ (with $i=1,\ldots ,n_{step}$) and the time increment is equal to $\Delta t_{i}=t_{i+1}-t_{i}$.

- The increment of total strain:
  (8) $$\Delta \epsilon_{i+1}^{to}=\Delta \epsilon_{i+1}^{el}+\Delta \epsilon_{i+1}^{kv}+\Delta \epsilon_{i+1}^{dash}=\Delta \epsilon_{i+1}^{el}+\Delta \epsilon_{i+1}^{v}$$
- The increment of elastic strain:
  (9) $$\Delta \epsilon_{i+1}^{el}=q_{k}C_{v}:\Delta \sigma_{i+1}\;\mathrm{with}\;q_{k}=\frac{1}{E_{k}}$$
- The increment of the two viscous strains:
  (10a) $$\Delta \epsilon_{i+1}^{kv}=\frac{\Delta t}{\tau_{kv}}\left[q_{kv}C_{v}:\sigma_{i+1}-\epsilon_{i+1}^{kv}\right]$$
  (10b) $$\Delta \epsilon_{i+1}^{dash}=\frac{\Delta t}{\eta_{dash}}C_{v}:\sigma_{i+1}$$

For the resolution of these equations, the quantities $\sigma_{i+1}$, $\epsilon_{i+1}^{kv}$ are evaluated in t+θΔt, i.e., $\sigma_{i+1}$ = $\sigma_{i}+\theta \Delta \sigma_{i+1}$. θ is taken between [0, 1], in this case, the implementation is fully implicit (θ =1). As a function *f* of strain increments, the implicit equations associated to the state variables are given by:

(11a) $$f\epsilon^{el}=\Delta \epsilon^{el}-\Delta \epsilon^{to}+\Delta \epsilon^{kv}+\Delta \epsilon^{dash}$$

(11b) $$f\epsilon^{kv}=\Delta \epsilon^{kv}-\frac{\Delta t}{\tau_{v}}\left[q_{kv}C_{v}:\sigma {{|}_{t+\theta \Delta t}-\epsilon^{kv}|}_{t+\theta \Delta t}\right]$$

(11c) $$f\epsilon^{dash}=\Delta \epsilon^{dash}-\frac{\Delta t}{\eta_{dash}}C_{v}{:\sigma |}_{t+\theta \Delta t}$$

Finally, these equations are solved by a Newton-type optimization method. The solving algorithm is presented in [42] and comprises several steps:

1. For a given time *t* in between $t_{i}$ and $t_{i+1}=t_{i}+\Delta t_{i+1}$, $\Delta \epsilon_{i+1}$ is known. At the beginning of the time step, the elastic stress increment is computed:
  (12) $$\Delta \sigma_{i+1}=K:\Delta \epsilon_{i+1}$$
  with K the fourth order elastic tensor explicitly given in Appendix B.
2. With this first estimation of the stress increment $\Delta \sigma_{i+1}$, the Newton minimization starts. The effective stress $\bar{\sigma }$ is computed (Equation (7)) at t+θΔt.
3. Nonlinear parameters are computed according to Equations (6a) and (6b).
4. The new increment $\Delta \sigma_{i+1}$ which minimizes the implicit system constituted by Equations (11a), (11b) and (11c) is determined.
5. If the minimization criterion is reached with the computed stress increment, then the minimization process stops, otherwise it returns to stage 2.
6. *i* is incremented and the process goes to stage 1.

#### 2.2.2. Implementation with MFront

The nonlinear creep model has been implemented using MFront tool (version 3.0) [42]. A Newton–Raphson algorithm with a numerical Jacobian made it possible to solve the system of nonlinear equation. Key implementation steps are summarized as follows:

1. At the beginning of the time step, the nonlinear parameters ($\eta_{kv}$, $\eta_{dash}$ and $E_{kv}$) are computed on each Gauss point.
2. Then, one must find the stress increment Δσ which verifies the structural equilibrium for a given strain increment Δϵ from the incremental relationship given in the Section 2.2.1.
3. Once the equilibrium is reached, the variables $\sigma_{i+1}$, $\epsilon_{i+1}^{el}$ and $\epsilon_{i+1}^{v}$ are updated.

## 3. Practical Case Studies

### 3.1. Samples of Bulk Epoxy Adhesive Subjected to Uniaxial Creep Loading

**Figure 2:**
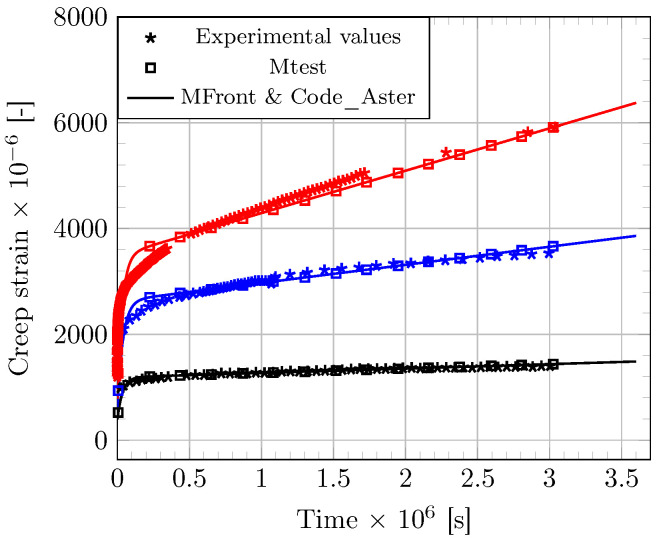
Comparison between theoretical and experimental strain for the bulk epoxy adhesive submitted to tensile creep at 5 MPa (black), 8 MPa (blue) and 10 MPa (red).

In a first stage, the constitutive law developed in the present work is compared with experimental data from a previous study [17], in which samples of bulk epoxy adhesive (Sikadur 30^®^ from Sika Company, Stuttgart, Germany) were subjected to sustained tensile loading. In this work, compliance master curves were constructed from series of a thermally stimulated tests according to the TTSP method. These master curves made it possible to obtain reference creep curves of the bulk epoxy adhesive over a 30-day period and for three load levels (i.e., at 5, 8 and 10 MPa). As the stress state is considered homogeneous within the sample, the problem can be simulated on a Gauss point in 0D, or alternatively on a unit cube. In this context, numerical simulations can be performed using Mtest module in 0D [42,43], or with the combined application of MFront and Code_Aster to an elementary FE cube (eight nodes, four Gauss points). A creep period of 30 days was considered in the simulations to facilitate comparison with experimental results. The input values of the model parameters were determined as follows: tensile tests were carried to evaluate the elastic modulus of the epoxy adhesive ($E_{k}$) [17], providing a mean value of 12.7 GPa very close to that reported in the technical data sheet [45]. A value of 0.3 was taken for the Poisson’s ratio ν of the epoxy adhesive in the vitreous state, as generally reported in the literature. The other model parameters were numerically identified from experimental data (Figure 2) and are reported in Table 1.

**Figure 3:**
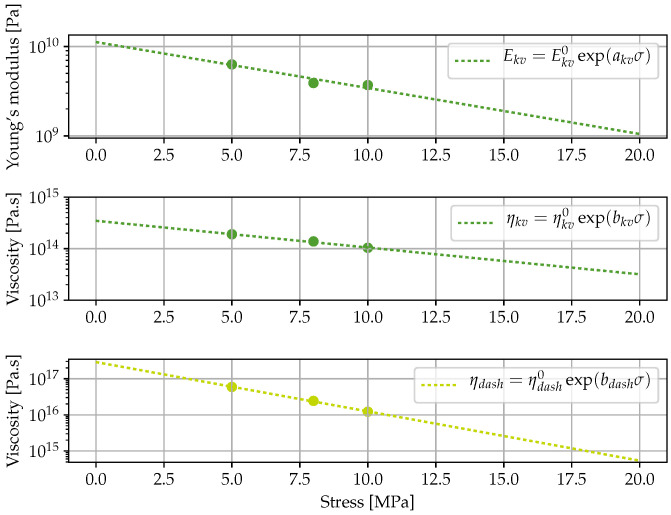
Comparison between the nonlinear functions identified in the present approach and the linear Burgers’ parameters identified at different stress levels by Houhou et al. [17].

Simulations provided by the two modelling methods (use of Mtest or combination of MFront-Code_Aster) are found consistent and are also fitting very well the experimental evidences (Figure 2). Moreover, in their work, Houhou et al. [17] fitted the creep master curves obtained at various stress levels using a linear Burgers rheological model, and then interpolated the variations of the model parameters over the stress range to manage the nonlinearities. The nonlinear functions developed in the present work are found to match very well with the parameter interpolations reported by Houhou et al. (Figure 3). These results support the validity of the nonlinear functions assumed in the present approach.

### 3.2. Structural Modelling

#### 3.2.1. Experimental Setup

**Figure 4:**
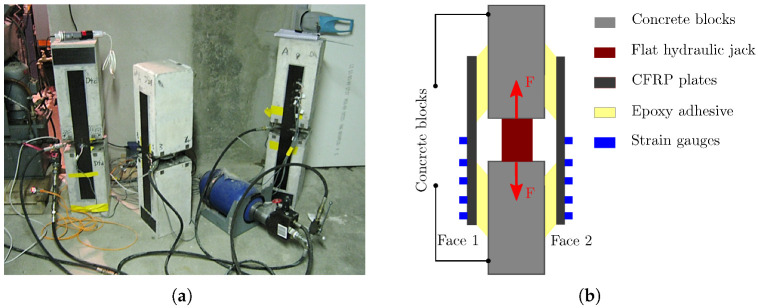
Creep test setup on double shear specimen previously proposed by Houhou et al. [17,45]. (**a**) Picture of the experimental setup, (**b**) principle of the test and loading procedure.

The second application is concerned with the modelling of a creep test on a double shear specimen previously developed by Houhou et al. [17,45]. The setup consists of two concrete blocks of dimensions 205 × 210 × 320 ${mm}^{3}$, connected by two CFRP (carbon fiber reinforced polymer) plates which are bonded to the lateral faces with the Sikadur 30 adhesive (here after referred to as “Face 1” and “Face 2”, see Figure 4). To ensure a perfect adhesion between composite plates and concrete, a specific surface treatment was applied. A step of abrasion with a grinding machine was performed to increase concrete’s roughness. In addition, CFRP plates were carefully degreased using a specific solvent. The mechanical loading was applied through a flat hydraulic jack installed at the center of the specimen, which pushes the concrete blocks and exerts a shear load on the concrete–CFRP joint. The hydraulic pressure was adjusted to provide an average shear stress of 0.6 MPa in the adhesive joints, which corresponds to 30% of the bond strength at 25 °C. This pressure was then maintained at a constant level during the entire creep test, thanks to a 10-liter accumulator connected to the hydraulic loop. In addition, the CFRP plates were instrumented by five strain gages bonded at their top surface (Figure 4). These sensors allow to evaluate the strain distribution along the adhesive joint, just after loading and during the creep phase as well. This creep experiment was conducted over one month. More details regarding this experimental setup can be found in [17,45].

#### 3.2.2. Description of the FE Model

**Figure 5:**
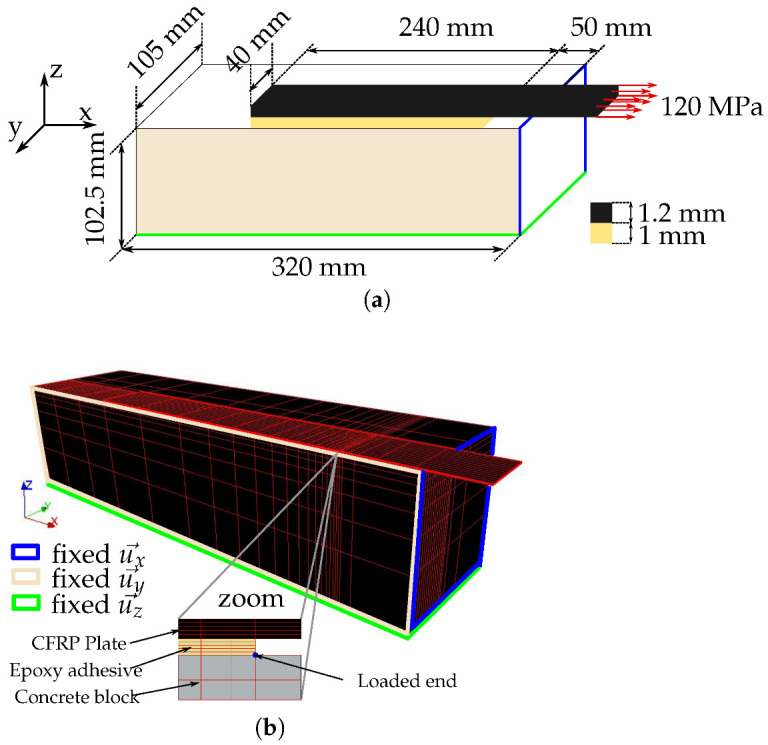
(**a**) Schematic representation and mesh of the double shear test, (**b**) associated FE mesh in Code_Aster.

Due to the symmetry of the system (geometry and boundary conditions), only one eighth of the system is modelled (Figure 5a shows the boundary conditions). The FE mesh (Figure 5b) consists of 10,189 linear hexahedrons. All component materials are assumed to exhibit a linear elastic behavior, except the epoxy adhesive. This assumption is motivated by the negligible creep response of the CFRP laminate in the longitudinal direction [46] and the low level of stress in concrete member (around 0.6 MPa). The CFRP composite is 1.2 mm thick and has an elastic modulus of 165 GPa and a Poisson’s ratio of 0.3. For the concrete material, values of 35 GPa and 0.3 were taken as the elastic modulus and the Poisson’s ratio, respectively. In the mesh, the adhesive joint is 1 mm thick and is represented by five layers of elements. Perfect bond is assumed between the different layers of concrete, adhesive and CFRP. Boundary conditions are imposed according to the scheme displayed (Figure 5a) and the sustained load is directly imposed on the cross section of the CFRP plate. Simulations were conducted considering a creep period of 30 days and different mechanical behaviors of the adhesive layer (i.e., elastic behavior, linear and nonlinear creep behaviors). Input parameters related to the adhesive layer that were implemented in the FE model are those previously reported in Table 1. In the case of an elastic behavior, only the parameter $E_{k}$ was considered and the others were set to zero. To describe linear creep, parameters $a_{kv}$, $b_{kv}$, $b_{dash}$ were taken equal to zero, whereas the whole set of parameters was implemented in the case of a nonlinear creep behavior.

#### 3.2.3. Confrontation between FE Model and Experimental Results

**Figure 6:**
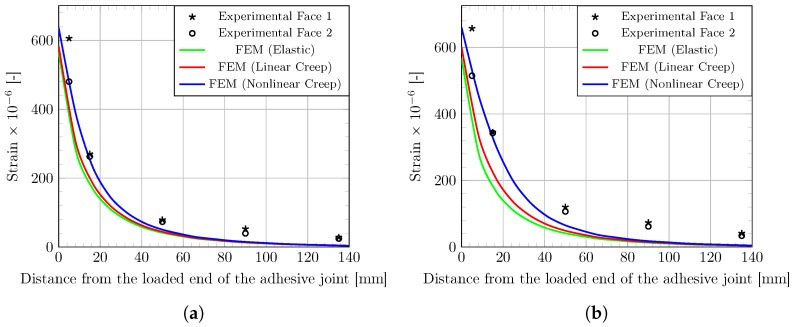
Axial strain ($\epsilon_{xx}$) profiles along FRP–concrete adhesive joint, (**a**) after the loading stage and (**b**) after an one-month creep period. Experimental data and simulations provided by the FE model considering different behaviors of the adhesive layer.

Figure 6 shows the strain profiles along the adhesive joint after the initial load application (Figure 6a) and after one month creep (Figure 6b). Both experimental data collected by the strain gages and theoretical profiles provided by the FE model are displayed in the graphs. Simulations are found globally consistent with the experimental evidences: strain values are around 600 × 10^−6^ at the loaded edge of the adhesive joint and fall to zero at 140 mm far from the edge.

However, the elastic and linear creep models tend to underestimate the strain values along the adhesive joint, both on the instantaneous and delayed responses. Differently, the nonlinear creep model captures well the strain profile from the loaded edge of the joint to a distance of 40–60 mm away from the edge, and then slightly underestimate the strain values over this distance. This can be explained both by the non-identification of the parameters on this experimental setup and by the model assumptions.

The modelling of adhesive shear creep was conducted using a creep model calibrated on tensile creep experiments. This simplified approach previously used by other authors [17,29] was adopted in the present work because experimental data of tensile creep tests performed on the same adhesive material were readily available from previous work (see Section 3.1). This attempt to model the shear creep behaviour of adhesive with such an assumption seems to be validated, in view of the good agreement between the simulations and the experimental data. In a further study, a more robust approach may be to calibrate the model directly from shear creep test data to capture the actual shear creep behaviour of the polymer adhesive. However, in practice, the implementation of these tests is more difficult than the creep test in tensile configuration.

#### 3.2.4. Sensitivity Analysis of the Elastic Parameters

**Figure 7:**
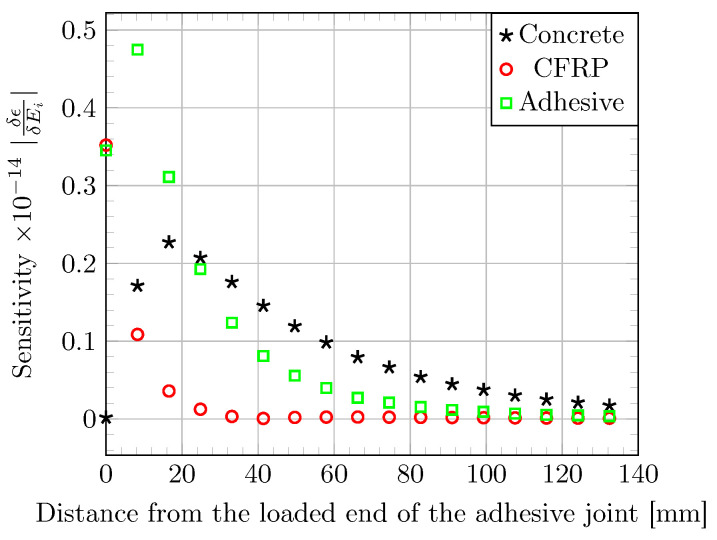
Sensitivity of the strain profile along the bonded joint to a 10% variation in the elastic moduli of the component materials, as evaluated using the elastic FE model.

To assess the influence of the properties of each component material (concrete, CFRP, adhesive) on the strain response of the bonded assembly, the effect of a finite variation in the Young’s modulus ($E_{i}$) of each component on strain profiles along the bonded joint is computed using the elastic FE model. The sensitivity of the strain profile to variations of 10% in the Young’s moduli of the various component materials is displayed in Figure 7. A variation in the stiffness of the CFRP material affects the strain profile over few millimeters near the loaded end and its influence decreases rapidly with the distance from the joint edge. In the same way, the strain profile is highly affected by a variation in stiffness of the epoxy adhesive near the joint edge, but the influence of the concrete’s modulus becomes predominant beyond a distance of 40–60 mm. This predominance may explain the gap between theoretical results and experimental data. In this study, there is no concern about concrete variability and concrete creep is neglected. These two assumptions may be the root of the differences. 

#### 3.2.5. Impact of Adhesive Creep on Stress Transfer Process

**Figure 8:**
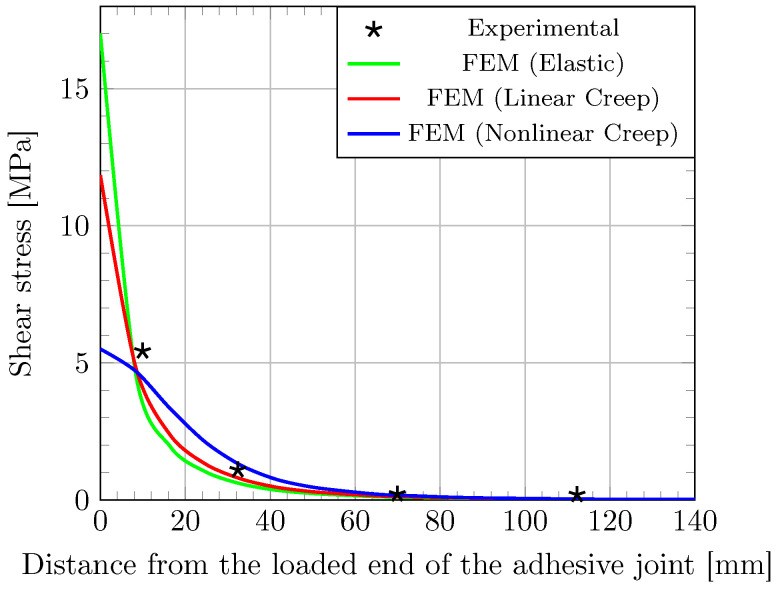
Shear stress profile along the bonded joint after a creep period of 30 days. Experimental data and simulations provided by the FE model considering different behaviors of the adhesive layer.

Finally, to investigate deeper the effect of creep on the load transfer process between the CFRP laminate and the concrete support, an analysis of the tangential stress distribution along the bonded joint (i.e., shear stress τ(x)) was carried-out. Tangential stresses were determined from experimental strain data, according to the procedure detailed in [17]. This method considers the equilibrium of an infinitesimal section of CFRP. As the distance between two strain gages is small, tangential stress is assumed constant over this length noted dx. Finally, a combination of the equilibrium equation and Hooke’s law makes it possible to express tangential stress versus strain (see Equation (13)).

(13) $$\left.\begin{array}{cc} \tau (x)dx & =-e_{cp}d\sigma \\ d\sigma & =E_{cp}d\epsilon \end{array}\right\}\Rightarrow \tau \left(x\right)=-e_{cp}E_{cp}\frac{d\epsilon }{dx}$$

with e${}_{cp}$ (m) and E${}_{cp}$ (Pa) the thickness and the Young’s modulus of the CFRP composite, respectively. Distribution of tangential stress along the lap joint determined by this method is plotted in Figure 8 (experimental data), together with the simulations provided by the FE model considering different types of behavior for the adhesive layer. As expected, stress distributions resulting from these various configurations are rather different. When examining the peak shear stress near the joint edge, values of 17.0, 11.8 and 5.50 MPa are obtained for the elastic, linear creep and nonlinear creep models, respectively.

As previously noticed for the strain distribution, the elastic FE model, that reflects the instantaneous behavior, is not able to capture the actual stress distribution and overestimate the peak value. In other words, it appears that creep induces a stress relaxation near the loaded end as previously reported by other researchers [15,31]. Differently, the stress profile predicted by the nonlinear creep model seems more coherent with experimental data, especially near the joint edge.

In addition, the anchorage length (which corresponds to the segment of the joint bearing at least 97% of the load applied to the assembly, according to Yuan [47]) estimated from the nonlinear creep simulation seems consistent with that from experimental data. As reported by others authors [18], the anchorage length is increased by the creep phenomenon.

## 4. Conclusions

This study has proposed a numerical investigation of the shear creep behavior of adhesive joints in the case of concrete structures reinforced by externally bonded CFRPs. This behavior is of particular importance near the loaded edge of the joint which is subjected to stress concentrations. In a first stage, constitutive equations were established using fully-3D formulation in order to describe the nonlinear creep response of the bulk adhesive. They were implemented using MFront tool and following an implicit method. The model parameters were identified by fitting creep strain data of bulk epoxy specimens which were available from a previous study. A FE model was then constructed by introducing the constitutive law in Code_Aster. In order to validate this numerical model, simulations were carried out according to the double shear creep test configuration proposed by Houhou et al. [17]. A good agreement was found with experimental evidence.

In addition, another interesting point revealed by those simulations is that the creep phenomenon occurring within the polymer adhesive modifies the stress distribution in the bonded joint and mitigates stress concentrations near the loaded edge (in comparison with the stress profile provided by a pure elastic model). This effect seems more effective when considering a nonlinear creep behavior of the polymer adhesive rather than a linear behavior. Furthermore, the simulations suggests that creep leads to an increased transfer length. This feature should be taken into account in the design of bonded FRP reinforcements. Finally, to further investigate the ageing behavior and the creep of adhesive joints, it may be interesting to refine the proposed model by taking into account the impact of environmental conditions such as temperature, relative humidity and material ageing in the modelling process. Furthermore, most of time, externally bonded systems are applied after several years of service and concrete has already experienced most part of its creep potential (basic creep or drying creep). Nonetheless, it may be interesting to consider concrete creep in the modelling in order to improve the robustness of theoretical outcomes.

## Figures and Tables

**Table 1:** Identified set of parameters of the proposed nonlinear creep model, for the bulk epoxy adhesive, subjected to sustained tensile loading.

E${}_{\mathrm{kv}}^{0}$ (GPa)	$a_{\mathrm{kv}}$ (MPa${}^{-1}$)	$\eta_{\mathrm{kv}}^{0}$ (GPa s)	$b_{\mathrm{kv}}$ (MPa${}^{-1}$)	$\eta_{\mathrm{dash}}^{0}$ (GPa s)	$b_{\mathrm{dash}}$ (MPa${}^{-1}$)
10.8	−1.10 × 10^−1^	3.47 × 10^5^	−1.19 × 10^−1^	2.88 × 10^8^	−3.14 × 10^−1^

## Data Availability

Data available on request.

## Abbreviations

The following abbreviations are used in this manuscript:

EB	Externally Bonded
FE	Finite Element
FRP	Fiber Reinforced Polymer
RC	Reinforced Concrete
TTSP	Time Temperature Superposition Principle

## Appendix A. Unit Tensor of Elastic Compliance

(A1) $$C_{v}=\left(\begin{array}{cccccc} 1 & -\nu & -\nu & 0 & 0 & 0\\ -\nu & 1 & -\nu & 0 & 0 & 0\\ -\nu & -\nu & 1 & 0 & 0 & 0\\ 0 & 0 & 0 & \left(1+\nu \right) & 0 & 0\\ 0 & 0 & 0 & 0 & \left(1+\nu \right) & 0\\ 0 & 0 & 0 & 0 & 0 & \left(1+\nu \right) \end{array}\right)$$

with ν (-) the elastic Poisson’s coefficient.

## Appendix B. Unit Tensor of Elastic Stiffness

(A2) $$K=\left(\begin{array}{cccccc} 2\mu +\lambda & \lambda & \lambda & 0 & 0 & 0\\ \lambda & 2\mu +\lambda & \lambda & 0 & 0 & 0\\ \lambda & \lambda & 2\mu +\lambda & 0 & 0 & 0\\ 0 & 0 & 0 & 2\mu & 0 & 0\\ 0 & 0 & 0 & 0 & 2\mu & 0\\ 0 & 0 & 0 & 0 & 0 & 2\mu \end{array}\right)$$

with λ (Pa) and μ (Pa) the Lame’s coefficients.
